# The impact of repetitive long-duration water immersion on vascular function

**DOI:** 10.1371/journal.pone.0181673

**Published:** 2017-07-27

**Authors:** Erin E. Simmons, Elizabeth R. Bergeron, John P. Florian

**Affiliations:** Navy Experimental Diving Unit, Panama City, Florida, United States of America; University of Southampton, UNITED KINGDOM

## Abstract

While physiological responses to water immersion (WI) are well-studied, the vascular responses after WI are less understood. Fifteen male subjects performed six-hour resting thermoneutral water immersions (WI) at 1.35 atmospheres absolute for four consecutive days, with follow-up on the fifth day. Measurements included peripheral endothelial function and augmentation index (PAT, peripheral arterial tonometry), beat-to-beat blood pressure (BP, photoplethysmography), heart rate (HR), and plasma volume (PV) calculated from changes in hemoglobin and hematocrit. The reactive hyperemia index (RHI), a marker of peripheral endothelial function, increased with repeated immersions (p = 0.008). By WI2 and WI3, RHI increased 12% and 16%, respectively, compared to WI1 values, but no significant differences were detected between WI4 and WI1 for either measure. Absolute augmentation index (AI) increased by an average of 33% (p<0.001) and AI normalized for HR (AI@75) by 11% (p = 0.12) following each WI. PV decreased significantly by 13.2% following WI and remained 6.8% lower at follow-up compared to pre-WI. Systolic blood pressure significantly decreased by an average of 2.5% following each WI (p = 0.012). Compared to pre-WI HR, average post-WI HR decreased 4.3% lower (p<0.001), but increased overall by 8.2% over the course of repeated WI (p<0.001). Total peripheral resistance increased by an average of 13.1% following WI (p = 0.003). Thus, peripheral endothelial function increases after two days of WI, and PAT-derived measures of arterial stiffness increase transiently post-WI. Additionally, BP and PAT-derived endothelial function diverge from their usual associations with arterial stiffness (i.e. augmentation index) in the context of WI.

## Introduction

A number of physiological effects of diving have been studied in an attempt to better understand acute and long-term risks associated with both pressure and water immersion. Complete characterization of systemic effects of pressure and immersion is especially important for individuals who are concerned with safety and performance in association with diving (i.e., working, rescue, or military divers). Autonomic and hemodynamic responses to water immersion (WI) have been documented [1,2,3,4,5,6,7], as well as changes in fluid distribution and excretion [5,8,9,10,11]. Water immersion increases central blood volume and plasma volume (PV), resulting in immersion diuresis [12,13]. The removal of hydrostatic pressure following WI leads to hypovolemia [11], increased calf vascular resistance (CVR), and decreased stroke volume (SV), cardiac output (CO), systolic blood pressure (SBP), and leg blood flow [13,14]. Vascular responses following long-duration WI are not well-characterized, although it is plausible that endothelial function and vascular stiffness may be affected.

While the endothelial cells lining blood vessels are responsible for many functions, including secretion of regulatory factors and inhibition of cellular adhesion, the primary role of the endothelium is facilitation of flow-mediated dilation [15,16]. A healthy endothelium is able to respond to increases in shear stress by causing vasorelaxation, a relationship first tested and observed by Furchgott and Zawadzki [17]. Nitric oxide (NO) has been identified as the endothelium-derived relaxation factor (EDRF) that causes vasodilation as well as inhibits the vasoconstrictor endothelin[16,18]. Changes in shear stress affect endothelial nitric oxide synthase (eNOS) activation and subsequently, NO production; it is therefore plausible that the changes in blood volume associated with hypovolemia following water immersion may affect endothelial function by altering vascular shear stress, but this pathway has not been studied.

Endothelial regulation of vessel tone in response to the balance between vasodilating and vasoconstricting substances may be related to measures of arterial stiffness. A reduction in nitric oxide may increase the stiffness of smooth muscle underlying the arterial endothelial cells, reducing the endothelial responses to shear stress [16]. Structural and functional changes in vessel walls that increase stiffness result in an increase in pulse wave velocity [19]. The increase in velocity causes the reflected wave to reach the heart during systole, thus increasing systolic blood pressure. While carotid-femoral pulse wave velocity is the current clinical gold standard for assessing arterial stiffness, analysis of pressure waveforms can also estimate arterial stiffness. The augmentation index (AI) is obtained by dividing the difference between the first and second systolic peaks by the pulse pressure. Utilizing this measure, a negative AI indicates normal, healthy vasculature while a positive AI is indicative of vessels with increased stiffness. There is some evidence that diving and water immersion may present cardiovascular challenges that affect arterial compliance, pulse pressures, and wave reflections[20]; however, such a relationship has yet to be determined.

Vascular responses to diving, as characterized by increased pressure with or without WI, while breathing compressed gases have been examined. The depth and duration of a dive are known to affect the uptake of inhaled gas into tissue, and subsequent reduction of pressure can cause bubbles to form as excess gas comes out of solution. Breathing compressed gas and the resulting decompression stress may thus decrease endothelial function. This is evidenced by the significant increase in the number of endothelial microparticles (a marker of endothelial damage) observed following compressed gas dives at 283 kPa[21]. This notion is supported by research showing that the production of reactive oxygen species (ROS) causes a reduction in endothelial function by inhibition of NO-mediated vasoregulation [22,23,24,25], and both ROS and intravascular bubble formation can result in physical endothelial cell damage [21,23,26,27,28,29].

Although previous diving studies have shown a decrease in endothelial function after diving with associated decompression stress [21,27,30,31], it is possible that head-out or shallow head-in WI alone will have a different effect on endothelial function [32], especially over the course of repeated immersions. Likewise, the effects of WI and resulting hypovolemia on measures of vascular stiffness are also unclear. It is reasonable to hypothesize that following resting immersion, the combination of inactivity together with post-immersion hypovolemia could increase arterial stiffness. Therefore, the objective of this study was to quantify the changes in endothelial function and vascular stiffness parameters following repeated six-hour WI with surface-supplied air. Endothelial function was tested by reactive hyperemia peripheral artery tonometry (RH-PAT), which is a non-invasive measure of the ratio of the pulse wave amplitude signal during reactive hyperemia compared to baseline. We also measured blood pressure and resistance measures as these are related to vascular function responses.

## Methods

### Subjects

Fifteen healthy male military divers with an average of 7±3 years of diving experience participated in the study. All subject characteristics can be found in Table 1. Subjects were not taking any medications pertinent to the outcome of the study, had not used nicotine in any form for the previous six months, and were asked to refrain from taking any elective medications, herbal supplements, or training supplements during the experimental period. Subjects were prohibited from alcohol for two days prior, and caffeine and exercise one day prior to the experimental period, and subjects were prohibited from diving for at least 10 days prior to the start of the experiment. A medical screening was performed, including complete blood count, complete metabolic panel, lipid profile evaluation, urinalysis, physical examination, and determination of maximal oxygen consumption ($\dot{V}O_{2\text{ max}}$). This protocol was approved by the Navy Experimental Diving Unit Institutional Review Board. Subjects provided written, informed consent, and all procedures conformed to the Declaration of Helsinki.

**Table 1:** Subject characteristics.

Age, y	30 ± 6 (21–40)
Weight, kg	88 ± 10
Height, cm	178 ± 9
BMI, kg/m^2^	28 ± 2
($\dot{V}O_{2\text{ max}}$), ml/kg/min	52 ± 7
SBP, mmHg	123 ± 7
DBP, mmHg	75 ± 11
Total cholesterol, mmol/l	4.27 ± 0.75
Diving Experience, y	7± 3

Values are means ± SD. n = 15 subjects. BMI, body mass index; SBP, systolic blood pressure; DBP, diastolic blood pressure.

### Study design

**Fig 1:**
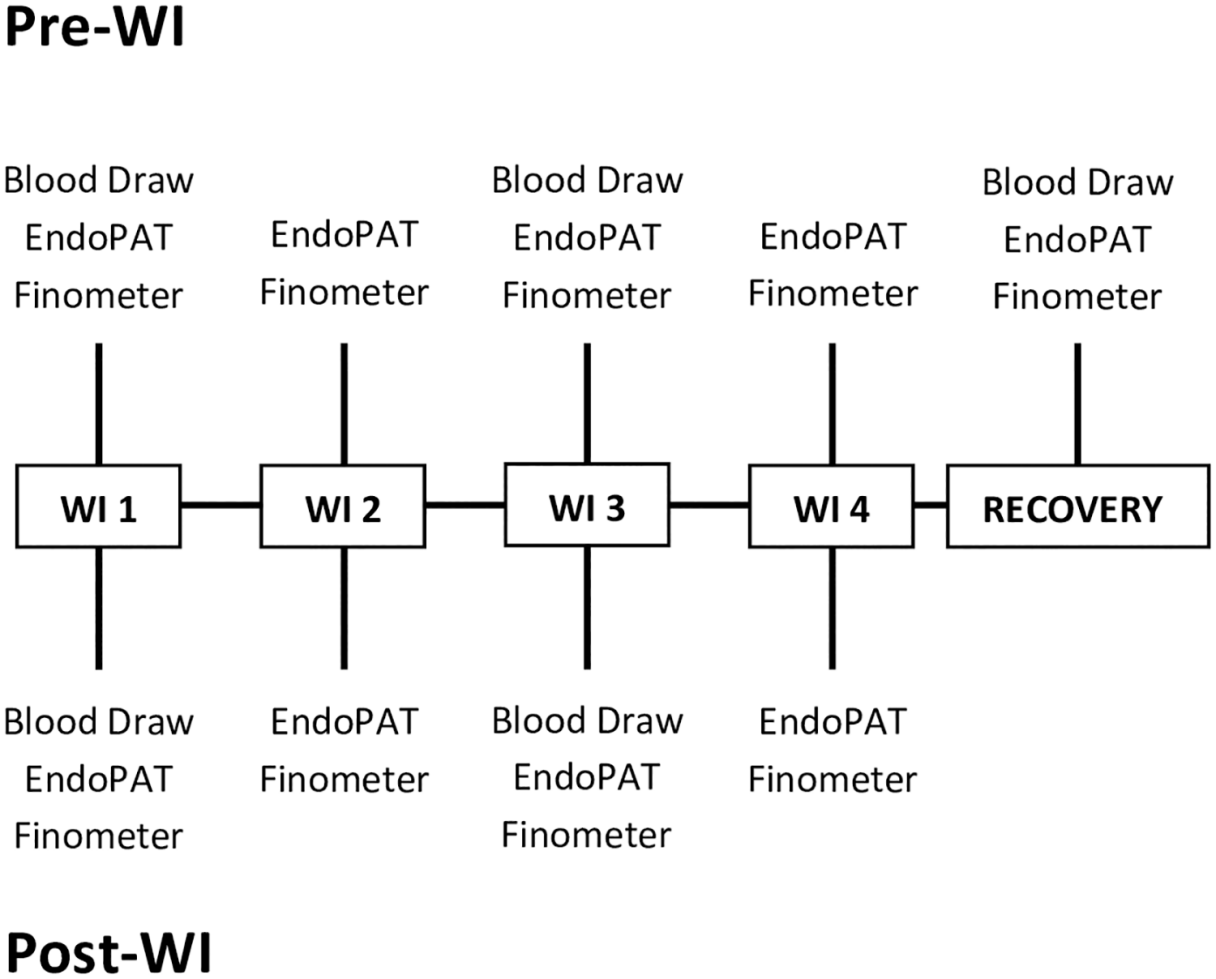
Water immersion and experimental timeline. Four consecutive water immersions (WI) were performed, immediately preceded and followed by blood draws (WI1, WI3, and Recovery), EndoPAT testing (WI1-4 and Recovery), and Finometer measurement (WI1-4 and Recovery).

The timeline of the experimental period is illustrated in Fig 1. Subjects were asked to abstain from food and drink (except water) for 2 hours prior to reporting to the laboratory. All physiological testing was performed before and after the 6-hour WI, and was conducted in a laboratory with air temperature maintained at 21–24°C. Subjects wore running shorts and t-shirts for each laboratory visit and during the dive. Pre-immersion measurements included the EndoPAT test and a blood draw to measure hemoglobin (Hb) and hematocrit (Hct) for ΔPV calculation. Subjects were then administered a standardized snack, emptied their bladder, and were weighed immediately prior to being immersed in the tank. They surfaced after hour 3 for a 10-min standardized lunch and returned to full immersion until hour 6. Post-WI weight was taken after the subject had emptied his bladder and was completely dried. Weight loss was calculated from the difference in pre- and postimmersion weight. Post-WI physiological measurements commenced after drying and weighing. This procedure was repeated for four consecutive days, and a follow-up (FU) test was conducted the morning of the fifth day.

### Water immersion

Each WI was conducted for 6 hours at the bottom of a 15 foot test pool with a thermoneutral water temperature of 32–33°C. Subjects were provided weights if necessary to maintain negative buoyancy, and were instructed to remain still and seated in a chair for the duration of WI. Subjects sat reclined so that their chest remained at a depth of 12–13 feet. They breathed surface-supplied air through a MK20 breathing apparatus (Aga mask, Interspiro), which delivers breathing gas at a pressure slightly greater than ambient pressure to minimize breathing resistance. At hour 3 of WI, participants were instructed to surface and stand on a platform with only head and shoulders out while eating a 2.2 MJ lunch containing 24% fat, 64% carbohydrate, 12% protein, and 500 ml of liquids, which included a supplementation shake (Ensure) and sports drink (Gatorade). After 10 minutes, participants returned to the bottom of the test pool until surfacing at hour 6.

### Vascular function measurement

Peripheral endothelial function and augmentation index were measured before and approximately 30 min after WI with the EndoPAT 2000 device (Itamar Medical, Caesarea, Israel) as previously described and reviewed [18,33,34,35]. RH-PAT measurements using the EndoPAT 2000 (Itamar Medical, Caesarea, Israel) have been shown to be highly reproducible [33,36,37], correlate well with brachial artery ultrasound measurement of flow-mediated dilation (FMD)[38,39,40], and detect the effects of acute interventions [21,41,42,43,44,45]. It is also recognized as an operator-independent method for both dynamic and diagnostic measures of vascular function [15,46,47,48]. The use of EndoPAT for finger tonometry is appropriate to measure endogenous NO-mediated vasodilation regulation since distal regions of limbs are major sites of sympathetic alpha adrenergic vasoconstrictor activity [15]. EndoPAT measures the change in peripheral arterial tone (PAT) signal at the fingertips in response to reactive hyperemia (RH-PAT) following upper arm occlusion. Endothelial function was measured using the reactive hyperemia index (RHI), which is determined from the ratio of post- to pre-occlusion PAT signal in the occluded arm relative to the same ratio in the non-occluded arm, and corrected for vascular tone of the occluded arm at baseline[37]. Augmentation index (AI) is a measure of wave reflections influenced by arterial stiffness, which is calculated by averaging multiple valid pulses during baseline in the occluded arm to determine the augmentation of the forward propagated wave by the returning reflective wave. This is accomplished by finding the systolic peak (P_1_) and the backward reflected peak (P_2_) and then using the formula: (P_2_-P_1_)/P_1_. Since the pulse waveform may be influenced by heart rate, AI can be corrected for heart rate by standardizing the measure to 75 bpm (AI@75). Study participants rested supine for 10 minutes before the start of the measurement period. Probes were placed on the index finger of both hands and a blood pressure cuff was applied to the non-dominant arm. Each recording consisted of a six-minute baseline period, a five-minute period of upper arm occlusion, and a five-minute post-occlusion measurement period. During occlusion, the blood pressure cuff was inflated to a suprasystolic pressure that did not exceed 300 mmHg. The RHI and AI values are automatically calculated by the EndoPAT system.

### Hemodynamic measurements

Beat-to-beat arterial pressure was measured during the EndoPAT protocol using photoplethysmography (Finometer, Finapres Medical Systems) on the ring finger of the dominant hand, and heart rate was measured using the EndoPAT. Total peripheral resistance (TPR), calculated by the Finometer as MAP / Finometer CO, was also recorded. Calibration of finger pressure to brachial artery pressure was performed using the manufacturer’s return-to-flow system. Beat-to-beat values of SBP, DBP, MAP, and TPR were averaged for approximately five minutes of the resting baseline period prior to occlusion of the non-dominant arm.

### Blood analysis

Blood was collected before and after WI and was immediately analyzed for Hb and Hct levels using the conductimetric method (Rapidpoint 405, Siemens). Blood draws were performed after ten minutes of seated rest, after which subjects lay supine during blood collection to prevent possible presyncopal symptoms which may occur after WI. The relative change in PV following WI was calculated from changes in Hb and Hct concentrations according to the Harrison modification of the Dill and Costill equation [49,50].

### Statistical analysis

A repeated measures analysis of variance (ANOVA) was conducted to determine the effect of WI on the dependent variables. SAS (Version 9.2) was used to run a 2 [Pre-WI, Post-WI] x 4 [Day1, Day2, Day3, Day4] mixed linear model ANOVA. When appropriate, a post-hoc Bonferroni-Holm correction was applied to identify differences between factors. Differences between FU and WI1 pre values were compared using a paired t-test.

## Results

### Weight loss and plasma volume

Mean weight loss after WI, adjusted for food and fluid intake was 1.80±0.14 kg (p<0.001). Plasma volume significantly decreased by 13.2% on average following each WI (p<0.001) and remained decreased by 6.8% at FU. There was no significant effect of WI day on the change in PV (Table 2).

**Table 2:** Responses to 6-hr water immersion.

*Variable*	*WI 1*	*WI 2*	*WI 3*	*WI 4*	*FU*	*Main* *Pre/Post*	*Main* *Day*	*Interaction* Pre/Post*Time
Weight (kg)								
*Pre*	88.3±2.8	88.7±2.9	87.9±2.7	87.7±2.8	87.5±2.8	<0.001*	<0.001*	0.572
*Post*	86.8±2.8	86.7±2.8	86.7±2.8	87.3±2.8				
dPV (%)								
*Pre*	-	-	-4.5±2.2	-	-6.8±1.9	<0.001*	0.397	0.312
*Post*	-13.0±1.8	-	-13.3±2.0	-				
SBP (mmHg)								
*Pre*	131±3	130±2	131±3	132±2	131±2	0.014*	0.169	0.425
*Post*	129±3	124±4	128±3	130±3				
DBP (mmHg)								
*Pre*	71±2	73±2	72±2	74±2	73±1	0.071	0.617	0.104
*Post*	77±2	73±3	72±3	74±2				
MAP (mmHg)								
*Pre*	92±3	92±2	93±2	94±2	92±2	0.580	0.455	0.291
*Post*	96±2	91±3	92±2	94±2				
TPR (dynes · s · cm^-5^)								
*Pre*	912±76	892±41	856±65	894±60	950±69	0.002*	0.064	0.253
*Post*	1110±80	1062±70	919±60	933±34				

Values are means ± SE for pre- and post-immersion variables. FU = follow-up, dPV = change in plasma volume, SBP = systolic blood pressure, DBP = diastolic blood pressure, MAP = mean arterial pressure, TPR = total peripheral resistance. Main, P-value of main effects of pre- and post-immersion and day of water immersion. Interaction, P-value of a two-way (pre/post * day) repeated-measures ANOVA interaction.

*P < 0.05.

### Endothelial function

**Fig 2:**
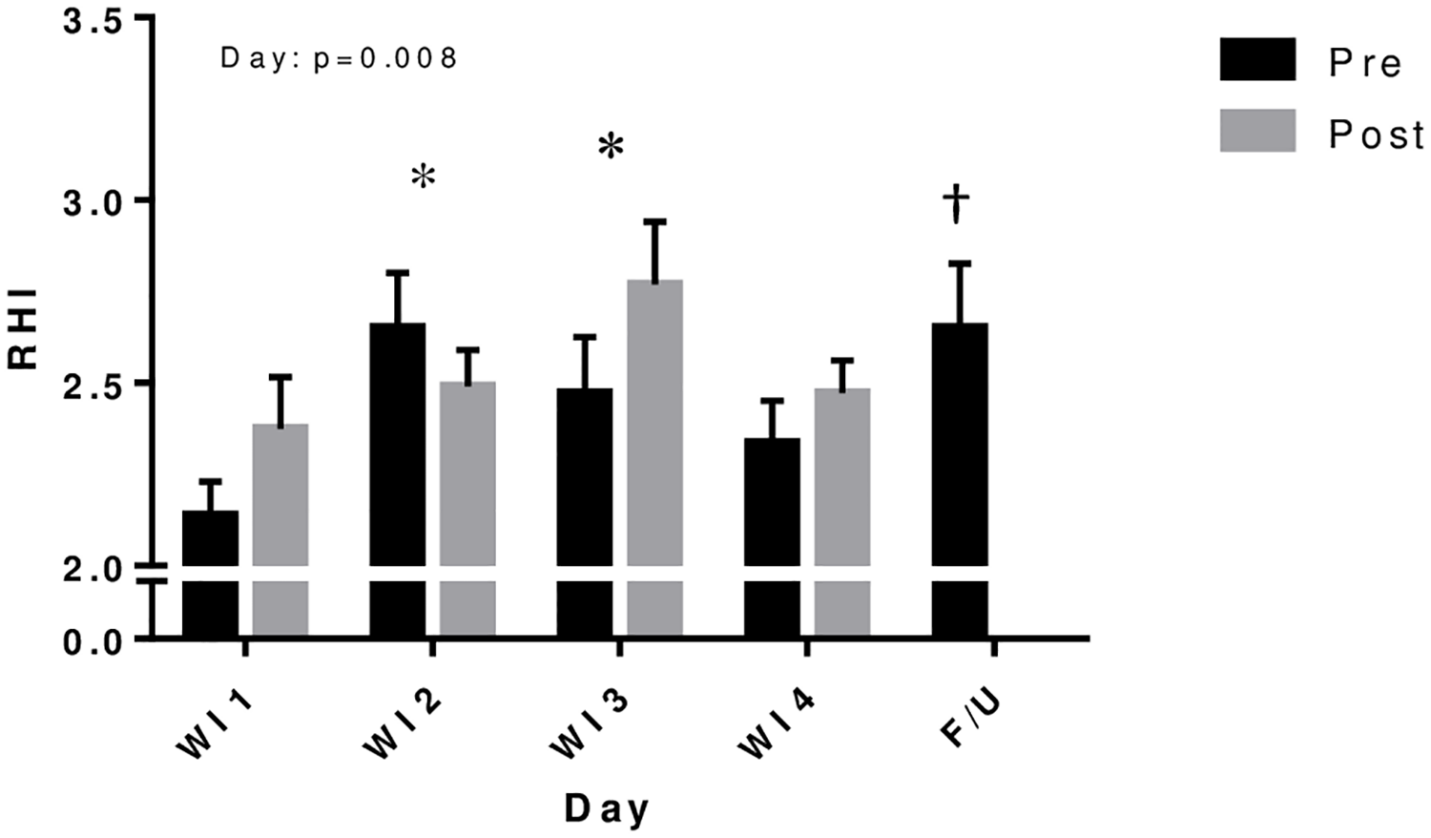
Peripheral endothelial function before and after repeated 6-h water immersions. Values are mean ± SE. *P<0.05 compared to water immersion 1 (WI1). †P<0.05 compared to WI1 pre. Immersion resulted in increased reactive hyperemia index (RHI) by the second and third days of WI. RHI during follow-up (F/U) remained elevated compared to baseline (WI1 pre).

The effect of WI day on RHI was significant (p = 0.008) and post-hoc analysis revealed a significant increase of 12.3% (p<0.01) on WI2 and 16.1% (p<0.01) on WI3 compared to WI1; WI4 was not significantly different from baseline values on Day 1 (Fig 2). There were no significant differences in RHI between pre- and post-WI measurements, but FU RHI was significantly higher than that at baseline (WI1 pre, p<0.005).

### Arterial stiffness parameters

**Fig 3:**
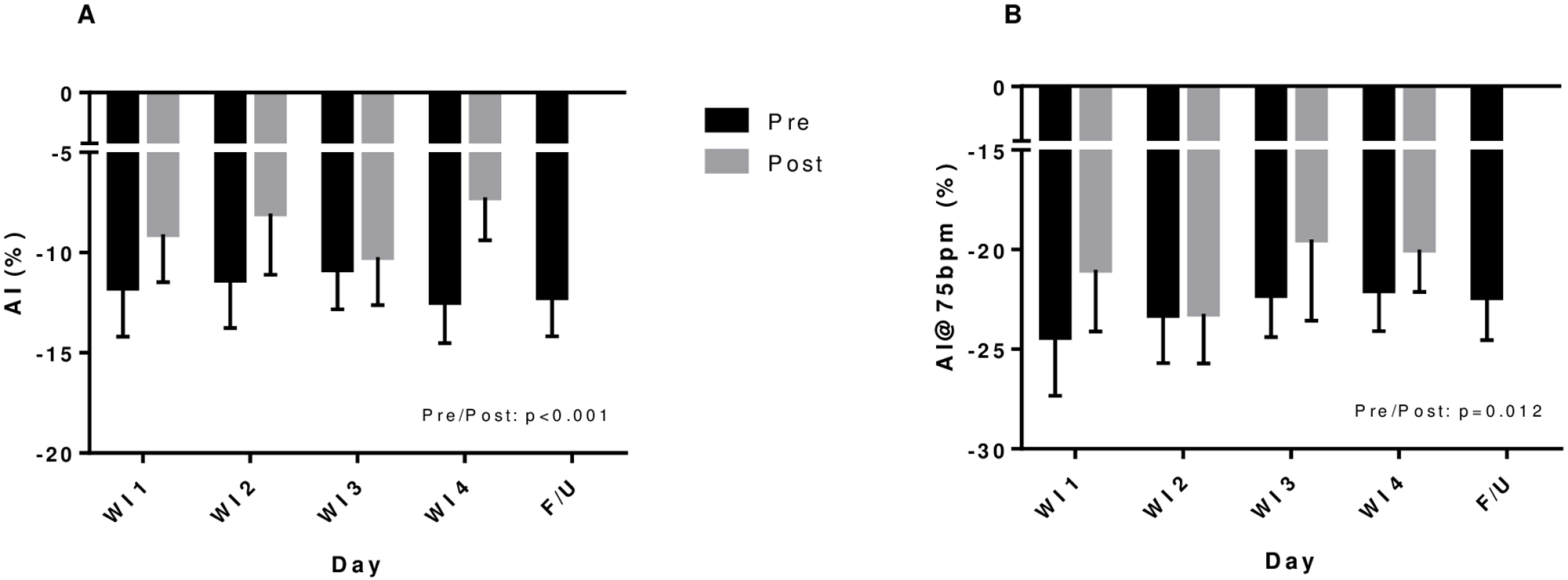
Arterial stiffness parameters before and after repeated 6-h water immersions. Values are mean ± SE. *P<0.05 compared to water immersion 1 (WI1). Increases in augmentation index (AI—A) and AI corrected for heart rate (AI@75—B) were seen following WI but there was no cumulative effect of repeated WI.

AI was significantly increased by an average of 33.3% following WI (p<0.001). There were no cumulative effects from consecutive days of WI (Fig 3A). When normalized for heart rate (AI@75), AI still showed a significant 10.7% increase post-WI (p = 0.012, Fig 3B). AI was not significantly different at FU compared to baseline (WI1) measures.

All interaction effects of the dependent variables were non-significant (Table 2).

### Blood pressure, heart rate, and resistance

**Fig 4:**
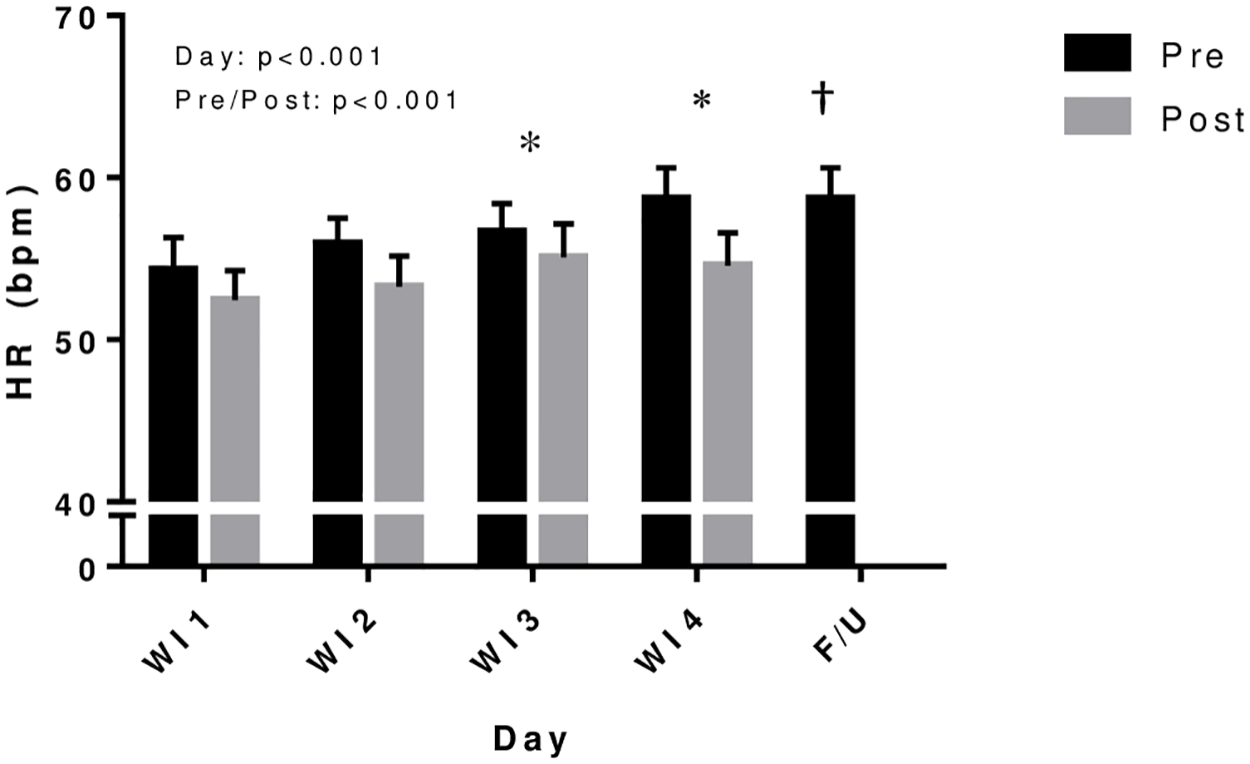
Heart rate before and after repeated 6-h water immersions. Values are mean ± SE. *P<0.05 compared to water immersion 1 (WI1). †P<0.05 compared to WI1 pre. Heart rate was lower post-WI but increased over the course of repeated immersions. Heart rate during follow-up (F/U) remained elevated compared to baseline (WI1 pre).

On average, SBP significantly decreased by 2.5% following WI (p = 0.012), but no significant differences in diastolic or mean arterial pressures were observed (Table 2). As shown in Fig 4, heart rate was 4.3% lower after WI on average (p<0.001), but showed a significant increase of 5.9% (p<0.002) by WI3 and 8.2% (p<0.001) by WI4 compared to WI1 (p<0.001), and remained elevated during FU compared to baseline (WI 1 pre). Total peripheral resistance (TPR) increased an average of 13.1% following WI (p = 0.003) with no significant effect of WI day (Table 2).

## Discussion

This study is the first to our knowledge to examine the effect of long, repeated water immersions on post-immersion RHI and AI, measures of endothelial function and arterial stiffness. RHI-PAT significantly increased by the third day of WI, but returned toward baseline levels on the fourth day. On the other hand, WI acutely increased AI after each WI, though this effect seemed to be transient as there was no change in AI detected pre-immersion for any WI days. Interestingly, blood pressure and endothelial function diverged from their usual associations with measures of arterial stiffness. These findings thus reveal atypical effects of WI on post-immersion vascular function.

This study contributes novel information related to diving and immersion physiology by separating out immersion effects from those caused by diving exposures with decompression stress. Previous studies have found that breathing air at depth resulted in decreased endothelial function, likely due to production of reactive oxygen species as well as bubbles in the circulation. Decreases in endothelial function measured by FMD were found following both dry and open-sea single dives for 80 minutes at 280 kPa and 30 minutes at 400 kPa, respectively, but these decreases in function were not correlated with bubble formation [27,31]. Differences may also exist depending on which breathing gases are utilized. In a previous study by Madden et al. [21], post-dive measures of endothelial function were significantly lower after dry, 60-minute air dives at 283 kPa compared to the same dive breathing 100% oxygen. Future studies should investigate whether RHI exhibits the same pattern with repeated oxygen WI as it does for WI while breathing air.

While endothelial function might be expected to decrease in response to the repetitive water immersions based on such previous diving studies, we recognized that there could be distinctive results for immersion without decompression. Indeed, our study showed that peripheral endothelial function actually improved from baseline by the second day of WI. We hypothesize that chronic exposure to increased arterial stiffness post-WI may lead to increased shear stress following each immersion. This could potentially increase NO production and cause an increase in RHI over the course of repeated immersions. An investigation of rehabilitation programs utilizing WI did in fact detect an increase in resting plasma concentration of the NO metabolite, nitrate, following five, 50-minute immersions per week for 3 weeks, suggesting upregulated endothelial function with chronic WI [32]. This may indeed be a chronic exposure effect, considering that a study measuring endothelial function in response to reactive hyperemia during WI found no change in plasma NO metabolites [51]. Future studies should further investigate the mechanisms behind the observed increase in RHI after repeated immersions, since a previous study found that FMD decreased on the third dive day in a series of repetitive dives [52]. While there is apparently a very different effect on endothelial function with diving compared to WI, it seems that there may be a consistent timing threshold (~2–3 days) for changes in endothelial function to take place. This observation should be tested further, and mechanisms by which an adaptive effect might occur should be explored.

Endothelial cell signaling has the potential to affect arterial stiffness via eNOS stimulation and NO production, and increased stiffness may promote a decline in eNOS activity and worsen endothelial cell functioning [53]. Though they have the potential to exert effects on each other, endothelial function and arterial stiffness are not necessarily linked [54]. Similarly, while chronic arterial stiffness is typically related to hypertension, there is evidence that measures of arterial stiffness and blood pressure can diverge acutely. A study of head-out WI found that blood pressure was significantly reduced while arterial stiffness increased [55]. Lazar et al. [55] proposed several factors that could potentially cause decreased BP with WI, including increased NO release and atrial natriuretic peptide, and decreased renin activity and plasma norepinephrine levels [50]. It is possible that the observed transient effects of WI on measures of arterial stiffness are not strong enough to affect blood pressure or endothelial function in ways previously determined in clinical studies of chronic cardiovascular states. Chronic increased stiffness generally contributes to increases in systolic blood pressure since the reflected wave reaches the heart faster and at systole, driving up systolic pressures. The acute increase in stiffness during immersion may not be enough to counteract the drop in systolic blood pressure caused by immersion-induced hypovolemia.

Other extrinsic influences on arterial stiffness include shear stress, angiotensin, and sympathetic neurohormones [53]. It is unlikely that traditional factors affecting arterial stiffness, such as increased collagen, decreased elastin, or deposition of sulfates, proteoglycans, or fibronectins, would act over the short time periods in this study. The lack of a significant effect of the WI day provides evidence that chronic structural changes did not occur over the course of the four days of WI, an observation supported by a previous study that found no tolerance effect of recent or repetitive head-out WI on changes in AI during immersion[50]. The transient increase in AI could be in response to reduced blood flow and inactivity during immersion, as well as post-immersion hypovolemia. Since previous studies have found that exercise has an acutely positive effect on compliance and stiffness, it is possible that inactivity and hypovolemia could have the opposite effect. Previous studies have shown that short-term inactivity results in greater arterial stiffness [56], and that blood volume and blood flow are reduced to extremities following immersion [14]. Additionally, increased extremity vascular resistance seen in our previous study may be linked to post-immersion peripheral arterial stiffness[14].

Previous studies have documented changes in arterial stiffness following both acute and chronic interventions, and acute changes have been detected in response to many stimuli other than diving or immersion [57,58,59,60,61]. Acute aerobic exercise (rowing) caused stiffness to decrease[62], and acute sprint interval exercise caused increased compliance but no change in brachial artery stiffness [63]. In both studies, however, chronic training resulted in decreased compliance and increased arterial stiffness. In another study [64], trained endurance athletes with higher $\dot{V}O_{2\text{ peak}}$ were found to have decreased arterial stiffness (AIx@75) compared to age-matched controls. Intensity of exercise may also affect arterial stiffness, which was shown to decrease significantly after high intensity interval exercise but not moderate continuous exercise [65]. Conversely, short-term bed rest (five days) resulted in decreased FMD and increased arterial stiffness [56].

Future work should identify the cause and time course of the transient increase in arterial stiffness parameters following WI. Although exercise and immersion appear to have opposite effects on arterial stiffness, the observation that some forms of chronic exercise tend to increase arterial stiffness begs the question of whether or not chronic diving or WI responds in a similar fashion. A recent study of female pearl divers suggests that chronic free-diving decreases arterial stiffness, but only compared to sedentary age-matched females; no difference was observed in arterial stiffness when compared to active age-matched females [20]. Taken with the observation that inactivity promotes increased arterial stiffness[56], there may be a greater effect of inactivity on arterial stiffness during our study than the immersion itself. Studies employing exercise during WI or an inactive time control condition on the surface would be able to determine whether it is the immersion or inactivity that contributes to the transient increase in arterial stiffness parameters that we observed.

While this study characterized the vascular response to repeated, long-duration WI, more research is needed to outline the mechanisms by which these responses occurred. The results of this study are limited to endothelial function and arterial stiffness, as measured by EndoPAT, in the context of resting WI while breathing surface-supplied air at 130 kPa. The effects of breathing oxygen or exercising during WI have yet to be determined. It is also important to note that AI has been questioned as a measure of arterial stiffness, as the characteristics of pulse waves can be determined by a variety of factors including small and large artery compliance, left ventricular function, artery elasticity, wave velocity, and heart rate (though a measure of AI normalized to heart rate is also presented here). Despite these limitations, we conclude that four consecutive days of six-hour WI while breathing air caused a transient increase in arterial stiffness parameters following each WI as well as an increase in endothelial function on the second day. Changes in systolic blood pressure also diverged from changes in arterial stiffness, which is a deviation from typical clinical findings. The mechanisms contributing to our results can only be speculated, and future studies should seek to determine the mechanisms by which our observations occurred.
